# Remittent Fever

**Published:** 1845-11

**Authors:** 


*Remittent Fever.*—The treatment in these cases, says Dr. G. Bird, is exceedingly simple. First, remove the hair, wash the body daily all over, and put the patient on low diet; and after clearing the bowels with hyd. c. cret., or ol. ricini, give sodæ sesquicarb, until the state of the secretions is corrected, quinine then given acts almost like magic, curing the patient generally in a week.—*Guys Hospital Reports.—Ibid.*

